# MUST: Minimal Unsatisfiable Subsets Enumeration Tool

**DOI:** 10.1007/978-3-030-45190-5_8

**Published:** 2020-03-13

**Authors:** Jaroslav Bendík, Ivana Černá

**Affiliations:** 8 Johannes Kepler University, Linz, Austria; 9 University of Birmingham, Birmingham, UK; Faculty of Informatics, Masaryk University, Brno, Czech Republic

**Keywords:** Minimal unsatisfiable subsets, Unsatisfiability analysis, Infeasibility analysis, MUS, Diagnosis

## Abstract

In many areas of computer science, we are given an unsatisfiable set of constraints with the goal to provide an insight into the unsatisfiability. One of common approaches is to identify minimal unsatisfiable subsets (MUSes) of the constraint set. The more MUSes are identified, the better insight is obtained. However, since there can be up to exponentially many MUSes, their complete enumeration might be intractable. Therefore, we focus on algorithms that enumerate MUSes *online*, i.e. one by one, and thus can find at least some MUSes even in the intractable cases. Since MUSes find applications in different constraint domains and new applications still arise, there have been proposed several *domain agnostic* algorithms. Such algorithms can be applied in any constraint domain and thus theoretically serve as ready-to-use solutions for all the emerging applications. However, there are almost no domain agnostic tools, i.e. tools that both implement domain agnostic algorithms and can be easily extended to support any constraint domain. In this work, we close this gap by introducing a domain agnostic tool called MUST. Our tool outperforms other existing domain agnostic tools and moreover, it is even competitive to fully domain specific solutions.

### 1 Introduction

In various areas of computer science, we are given a set $C$ of constraints with the goal to determine whether the set is satisfiable, i.e. whether all the constraints can hold simultaneously. In the case where the set is shown to be unsatisfiable, we are often interested in analysing the unsatisfiability. Identification of minimal unsatisfiable subsets (MUSes) of $C$ is a kind of such analysis. A set $M \subseteq C$ is a MUS of $C$ iff $M$ is unsatisfiable and all proper subsets of $M$ are satisfiable. The more MUSes are identified, the better insight into the unsatisfiability of $C$ is obtained. However, the complete MUS enumeration is often intractable since

* This research was supported by ERDF "CyberSecurity, CyberCrime and Critical Information Infrastructures Center of Excellence" (No. CZ.02.1.01/0.0/0.0/16_019/0000822).

there can be up to exponentially many MUSes w.r.t. the number of constraints in $C$. Therefore, several *online* MUS enumeration algorithms (e.g. [3,29,22,1,25,10]) were proposed, i.e. algorithms that identify MUSes gradually, one by one, and thus identify at least some MUSes even in the intractable cases.

Various applications of MUSes arise for example in requirements analysis [4,6], during formal equivalence checking [15], proof based abstraction refinement [23], Boolean function bi-decomposition [12], circuit error diagnosis [21], type debugging in Haskell [30], or proof explanation in symbolic model checking [20]. The domain of the constraint sets ranges from Boolean formulas [23,14], over temporal logic formulas [4,6], to transition state predicates constraining transition systems [20]. Since the list of constraint domains where MUSes find an application is quite long and new applications still arise, there have been proposed several *domain agnostic* MUS enumeration algorithms (e.g. [3,22,9,7,10]). Such algorithms can be used in an arbitrary constraint domain, and thus theoretically serve as ready-to-use solutions for any constraint domain where MUSes might eventually find an application.

Unfortunately, there is no available *domain agnostic tool implementation* of the algorithms that would actually serve as a ready-to-use solution for an arbitrary constraint domain. Although the papers that present existing domain agnostic algorithms provide results of an experimental evaluation, it is often the case that the implementation is either not publicly available [4,3], or there is a hard-coded support for a particular constraint domain [10,20]. The closest to a domain agnostic tool is a tool by Liffiton et al. [22] where the authors implement their domain agnostic MUS enumeration algorithm MARCO. Their tool currently supports the SAT and the SMT domains and can be relatively easily extended to support also another constraint domains. However, our recent evaluation [8] of contemporary domain agnostic algorithms in various constraint domains has shown that the efficiency of the algorithms (including MARCO) varies a lot in different constraint domains. There is no silver bullet algorithm that would be efficient in all the domains. Thus, to deal with a particular constraint domain, one has to wisely choose from individual algorithms.

In this work, we present the first stable release of our domain agnostic tool, called `MUST`, for MUS enumeration. The tool implements several domain agnostic MUS enumeration algorithms and currently provides support for 3 constraint domains: SAT, SMT, and LTL. Moreover, due to a modular architecture of the tool, the tool can be easily extended to support another constraint domain: it requires only to implement an API for communication with a satisfiability solver for the constraint domain. We also provide a guidance on which algorithms are suitable for which kinds of input constraint systems.

To demonstrate the efficiency of our tool, we experimentally compare it to the tool by Liffiton et al. [22] in the SAT and SMT domains, and we show that our tool clearly wins in both the domains. Moreover, we also provide a comparison with two contemporary tools that are tailored to the SAT domain: `MCSMUS` [1] and `FLINT` [25]. The results show that `MUST` is competitive to the two domain specific solutions. Moreover in case of many benchmarks, MUST actually significantly dominates the other tools.

Finally, to advocate the practical applicability of our tool in industrial settings, we provide a use case from the area of requirements analysis. In particular, we have employed our tool in the European Unions Horizon 2020 project called AMASS. The project focused on development and verification of cyber-physical systems in the largest industrial markets including automotive, railway, aerospace, space, and energy. One of the verification tasks is to verify that requirements on the system are consistent, i.e., to ensure that there can be even built a system that satisfies the requirements. If the requirements are found to be inconsistent, an identification of minimal inconsistent (unsatisifable) subsets of the requirements helps to fix the conflicts among the requirements. Our tool has proved to be very efficient in dealing with this task.

### 2 Preliminaries

#### 2.1 Basic Definitions

We are given a set $C = \{c_1, c_2, \ldots, c_n\}$ of constraints such that each subset of $C$ is either *satisfiable* or *unsatisfiable*. The notion of satisfiability varies in particular constraint domains. We only assume that if a set $N$, $N \subseteq C$, is satisfiable then all subsets of $N$ are also satisfiable. Dually, if a set $K$, $K \subseteq C$, is unsatisfiable then all supersets of $K$ are also unsatisfiable. We will use $C$ to denote the input set of constraints throughout the whole paper.

**Definition 1 (MUS).** *A subset $N$ of $C$ is a minimal unsatisfiable subset (MUS) of $C$ if and only if $N$ is unsatisfiable and for all $c \in N$ the set $N \setminus \{c\}$ is satisfiable.*

Note that the minimality concept used here is set minimality, not minimum cardinality. Therefore, there can be MUSes with different cardinalities. Also, there can be up to exponentially many MUSes w.r.t. the number of constraints in $C$ (see the Sperner's theorem [28]).

**Definition 2 (critical constraint).** *Let $U$ be an unsatisfiable subset of $C$ and $c \in U$. The constraint $c$ is* critical *for $U$ if and only if $U \setminus \{c\}$ is satisfiable.*

Note that $U$ is a MUS of $C$ if and only if all constraints in $U$ are critical for $U$. Furthermore, if $c$ is critical for $U$ then $c$ has to be contained in every MUS of $U$.

*Example 1.* We illustrate the concepts on a small example. Assume that we are given a set $C$ of four Boolean satisfiability constraints: $c_1 = a$, $c_2 = \neg a$, $c_3 = b$, and $c_4 = \neg a \vee \neg b$. Clearly, the whole set is unsatisfiable as the first two constraints are negations of each other. There are two MUSes: $\{c_1, c_2\}$, $\{c_1, c_3, c_4\}$. As for the critical constraints, we can for example see that $c_1$ is the only critical constraint for $C$, and that $c_1, c_2$ are critical for $\{c_1, c_2, c_3\}$.

**Algorithm 1:** Domain Agnostic Shrinking

**input** : an unsatisfiable set $S$ of constraints
**input** : a set *crits* of constraints that are critical for $S$
**output:** A MUS of $S$
**for** $c \in S \setminus crits$ **do**
  **if** *not* `CheckSat`$(S \setminus \{c\})$ **then**
    $S \leftarrow S \setminus \{c\}$
**return** $S$

#### 2.2 Shrink

Let us now define an operation, called `Shrink`, that is used in our tool to identify individual MUSes.

– `Shrink(`$S$`, ` *crits*`)` takes an unsatisfiable subset $S$ of $C$ together with a set *crits* of constraints that are critical for $S$ and returns a MUS $S_{mus}$ of $S$.

We say that $S$ is *shrunk* into a MUS $S_{mus}$. The shrinking is maintained in in our algorithms as a black-box subroutine and thus can be implemented using any available single MUS extraction algorithm. Especially, we can implement the operation using a domain specific solution and thus indirectly exploit domain specific properties of particular constraint domains.

To shed more light on how a shrinking can be done, we describe in Algorithm 1 a domain agnostic single MUS extraction approach that forms the basis of many contemporary domain specific solutions. To find a MUS of $S$, the algorithm iteratively attempts to remove individual constraints in $S \setminus crits$ from $S$, checking each new set for satisfiability, and keeping only the changes that preserve $S$ to be unsatisfiable. The most expensive part of the shrinking are the satisfiability checks. In total, the algorithm performs $|S| - |crits|$ satisfiability checks. Domain specific algorithms (e.g. [5,24,1,19]) that are based on Algorithm 1 are often able to further reduce the number of performed satisfiability checks by exploiting domain specific properties of particular constraint domains.

#### 2.3 Unexplored Subsets

Our algorithms for MUS enumeration during their computation gradually *explore* satisfiability of individual subsets of $C$. The *explored* subsets are those, whose satisfiability is already known by the algorithm whereas *unexplored* subsets are those whose satisfiability is not determined yet. We use *Unexplored* to denote the set of all unexplored subsets of $C$. Recall that all subsets of a satisfiable set are also satisfiable. Thus, if a set $S$ is determined to be satisfiable, then not just $S$ but also all of its subsets become explored. Dually, if a set $U$ is determined to be unsatisfiable, then all supersets of $U$ become explored. We further classify unexplored subsets as follows:

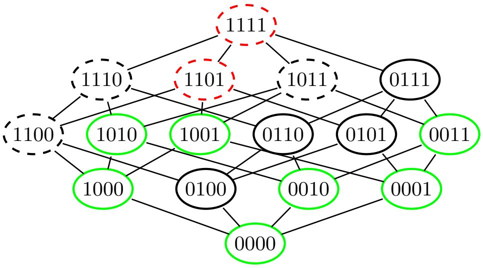

Fig. 1: Illustration of Example 2. We encode individual subsets of C as bit-vectors; for example, the subset $\{c_1, c_3, c_4\}$ is written as 1011.

**Definition 3 (Minimal Unexplored Subset).** *A set $S$ is a minimal unexplored subset, if $S$ is unexplored and for all $c \in S$ is $S \setminus \{c\}$ explored.*

**Definition 4 (Maximal Unexplored Subset).** *A set $S$ is a maximal unexplored subset, if $S$ is unexplored and for all $c \in C \setminus S$ is $S \cup \{c\}$ explored.*

Details on how we actually store, maintain, and use unexplored subsets are described later in Section 4.2. Here, we conclude by defining the concept of *minable critical* constraints:

**Definition 5 (minable critical).** *Let $N$ be an unsatisfiable subset of $C$ such that $N \in$ Unexplored, and let $c \in N$. The constraint $c$ is a* minable *critical constraint for $N$ if $N \setminus \{c\} \notin$ Unexplored.*

*Example 2.* Let us illustrate the concepts on an example. Assume that we are given the same set of four constraints as in Example 1: $c_1 = a$, $c_2 = \neg a$, $c_3 = b$, and $c_4 = \neg a \vee \neg b$. Fig. 1 shows a possible state of exploration of the power-set. Satisfiable subsets are drawn with a solid border and unsatisfiable ones with a dashed border. There are 2 explored unsatisfiable subsets (red color), 7 explored satisfiable subsets (green color), and 7 unexplored subsets (black color). There are two minimal unexplored subsets: $\{c_2\}$ and $\{c_1, c_3, c_4\}$, and three maximal unexplored subsets: $\{c_1, c_2, c_3\}$, $\{c_1, c_3, c_4\}$ and $\{c_2, c_3, c_4\}$. As for the minable critical constraints, we can for example see that $c_2$ is minable critical for the set $\{c_1, c_2, c_3\}$, and that all constraints are minable critical for the set $\{c_1, c_3, c_4\}$.

### 3 Implemented Algorithms

Our tool currently implements three domain agnostic algorithms for online MUS enumeration: MARCO [22], TOME [7], and ReMUS [9]. MARCO was originally developed by Liffiton et al. [22]; the other two algorithms are originally ours. All the three algorithms are based on a common scheme that we call *seed-shrink scheme*. In this section, we first describe the base scheme and then briefly comment also on the individual algorithms.

**Algorithm 2:** Seed-Shrink Scheme

**input :** an unsatisfiable set $C$ of constraints
**output:** All MUSes of $C$
$\mathit{Unexplored} \leftarrow \mathcal{P}(C)$
**while** there is a seed **do**
  $S \leftarrow$ find a seed
  $\mathit{crits} \leftarrow$ collect minable critical constraints for $S$
  $S_{mus} \leftarrow \texttt{Shrink}(S, \mathit{crits})$
  $\mathit{Unexplored} \leftarrow \mathit{Unexplored} \setminus \{T \mid T \subset S_{mus} \text{ or } S_{mus} \subseteq T \subseteq C\}$
  **output** $S_{mus}$

#### 3.1 Seed-Shrink Scheme

The *seed-shrink scheme* is shown in Algorithm 2. The computation starts by initializing the set *Unexplored* to $\mathcal{P}(C)$, i.e. all subsets of $C$ are initially unexplored. Subsequently, the scheme iteratively identifies all MUSes of $C$. Each iteration starts by finding a so called *seed*, i.e. an unexplored subset that is unsatisfiable. Subsequently, the set *crits* of all constraints that are minable critical for the seed are collected and the shrinking procedure is used to find a MUS of the seed. The iteration is concluded by marking all subsets and supersets of the MUS as explored (the subsets are necessarily satisfiable, and the supersets are unsatisfiable). The computation terminates once there is no more seed.

The scheme does not specify how to find a seed; this part differs for individual algorithms implementing the scheme. In general, to find a seed, the algorithms check several unexplored subsets for satisfiability and reduce the set *Unexplored*. The difference between the algorithms is in *which* and *how many* subsets they check, and *how large* is the resultant seed. In general, the smaller the seed is, the easier is to shrink it. On the other hand, unsatisfiable subsets are naturally more concentrated among the larger subsets, thus looking for a seed among small unexplored subsets might come with the price of checking many unexplored subsets for satisfiability. Individual seed-shrink algorithms make a different trade-off between the size of identified seeds and the number of satisfiability checks that are performed to identify the seeds. In some constraint domains, it is worth to find a small seed even if it requires performing many satisfiability checks, and in other constraint domains the situation is exactly the opposite. The optimal choice of a seed-shrink algorithm thus differs for individual constraint domains.

**MARCO** [22] searches for a seed $S$ among the maximal unexplored subsets and often performs only few satisfiability checks to identify a seed. Since maximal unexplored subsets are usually very large, the seeds identified by MARCO are generally hard to be shrunk. Yet, in some constraint domains, such as SAT and SMT, the size of the seed has just a negligible effect on the complexity of the shrinking. In particular, in the SAT and SMT domains, contemporary satisfiability solvers can extract an *unsat core* of the seed $S$, i.e. unsatisfiable, yet not necessarily minimal, subset of $S$. The extraction comes with almost no overhead compared to an ordinary check for satisfiability, and the unsat core is usually very close, in terms of cardinality, to a MUS of $S$. Thus, instead of shrinking the whole $S$, the unsat core is passed to the shrinking procedure.

**TOME** [7] identifies seeds iteratively as follows. Each iteration of the algorithm starts by picking a minimal unexplored subset $N_1$ and a maximal unexplored subset $N_p$ such that $N_1 \subseteq N_p$. Subsequently, TOME builds a chain $N_1 \subset N_2 \subset \cdots \subset N_p$ of unexplored subsets. Such a chain necessarily either contains only unsatisfiable subsets, only satisfiable subsets, or it contains an element $N_i$ such that $\forall j$, $1 \leq j < i$, is $N_j$ satisfiable and $\forall k$, $i \leq k \leq p$, is $N_k$ unsatisfiable. In the first case, it is guaranteed that $N_1$ is a MUS. In the second case, the chain does not give us any seed. Finally, in the third case, TOME finds $N_i$ using binary search (which takes only $\mathcal{O}(\log_2 p)$ satisfiability checks). Subsequently $N_i$ is used as a seed for the shrinking procedure and shrunk into a MUS.

There are no guarantees on distribution of satisfiable and unsatisfiable subsets on the chain, since the subsets are unexplored. In the best case, where $N_1$ is unsatisfiable, TOME identifies a MUS using just a single satisfiability check. In the worst case, the whole chain is satisfiable and TOME has to build another chain. Based on our experience, TOME on average performs more satisfiability checks to find a seed than MARCO does, but the seeds are much smaller than in the case of MARCO. Thus, TOME is efficient especially in constraint domains where the size of the seed highly affects the complexity of the shrinking.

**ReMUS** [9] is based on the following observation: if $C$, $C^k$, and $M$ are unsatisfiable sets such that $C^k \subseteq C$ and $M$ is a MUS of $C^k$, then $M$ is necessarily also a MUS of $C$. Note that the smaller $C^k$ is the smaller seeds are in $C^k$. ReMUS tends to identify $C^k$ that is very small, yet contains many MUSes, and searches for seeds in $C^k$. In particular, the very first seed $S$ is found among the maximal unexplored subsets of $C^0 = C$ and then shrunk to a MUS $S_{mus}$. To find a next seed, ReMUS chooses $C^1$ such that $S_{mus} \subseteq C^1 \subseteq S$, and searches for a seed $S^1$ among maximal unexplored subsets of $C^1$. If a seed $S^1$ is identified, then it is again shrunk to a MUS $S^1_{mus}$ and again used to reduce the search space, i.e. the a next seed $S^2$ is searched for in a set $C^2$ such that $S^1_{mus} \subseteq C^2 \subseteq S^1$. The search space reduction is recursively repeated as long as possible. Once the current search space is completely explored, ReMUS backtracks from the recursion and searches for a seed on the previous recursion level. Moreover, ReMUS employs several heuristics to pre-emptively backtrack from a search space that contains a lot of unexplored subsets but only few MUSes.

The larger the input set $C$ of constraints is, the more extensive recursive reduction is possible, and thus the smaller seeds can be found. We recommend to use ReMUS, rather than MARCO or TOME, if the input constraint set contains at least hundreds of constraints and hundreds of MUSes, no matter what the constraint domain is.

For a more elaborated description of the three algorithms, please refer to the original papers [22,7,9] or to our recent work [8] where we have experimentally compared the algorithms in various constraint domains.

## 4 Architecture of the Tool

Our tool is implemented in C++ and is available under the MIT license at:

https://github.com/jar-ben/mustool

The tool consists of six logical components: *SatSolver*, *Explorer*, *Master*, *Algorithms*, *Heuristics*, and *Initializer*. In the following section 4.1 we provide a brief description of the individual components. Subsequently, in Sections 4.2 and 4.3 we provide a more detailed description of *Explorer* and *SatSolver*. Finally, in Section 4.4, we give instructions on how to install and use our tool.

### 4.1 Logical Components

**SatSolver** *SatSolver* (declared in *SatSolver.h*) is the only domain specific part of our tool. It provides the functionality for checking sets of constraints for satisfiability, and implements the shrinking procedure. Also, *SatSolver* copes with parsing the input set of constraints (provided by the user) and exporting the identified MUSes in particular domain specific formats. A more detailed description of *SatSolver* is provided in Section 4.3.

**Explorer** *Explorer* (declared in *Explorer.h*) maintains the set *Unexplored* of all unexplored subsets and handles related operations including: marking sets as explored, obtaining unexplored subsets, and mining critical constraints based on the set *Unexplored*. For more information, see Section 4.2.

**Master** *Master* (declared in Master.h) is the coordinator of the whole computation. In particular, it holds an instance of Explorer and an instance of SatSolver and provides wrappers for calling their methods. Moreover, it runs a MUS enumeration algorithm that is specified by the user via a command line argument (see below).

**Algorithms** The algorithms MARCO [22], TOME [7], and ReMUS [9] are declared in Master (Master.h) and implemented in marco.cpp, tome.cpp, and remus.cpp, respectively. All calls to SatSolver and Explorer are made via the wrappers defined in Master. This means that any improvement to Explorer and especially to SatSolver (i.e. a more efficient shrinking procedure or satisfiability solver) is immediately reflected by all the algorithms.

**Heuristics** There are several heuristics that are bound to the wrappers defined in Master, and thus can be exploited by all the three algorithms. For example, in the wrapper for invoking the shrinking procedure, we provide two heuristics for computing critical constraints for the set that is being shrunk. One of the two heuristics uses Explorer to compute critical constraints based on the set *Unexplored*. The other heuristic uses SatSolver to obtain additional critical constraints that cannot be mined from *Unexplored*.

**Initializer** *Initializer* (implemented in main.cpp) parses the command line arguments provided by the user, and creates, sets-up, and runs the Master.

#### 4.2 Explorer

Since there can be up to exponentially many unexplored subsets w.r.t. the number of constraints in $C$, it is intractable to represent them explicitly. Instead, we adopt a symbolic representation that was first proposed by Liffiton et al. [22] and subsequently used in many other works (e.g. [1,20,10]).

Given a set $C = \{c_1, c_2, \ldots, c_n\}$ of constraints, we introduce a set $X = \{x_1, x_2, \ldots, x_n\}$ of Boolean variables, and maintain two Boolean formulas, $map^+$ and $map^-$, over $X$ such that each model of $map^+ \wedge map^-$ corresponds to an unexplored subset and vice versa. The formulas are maintained as follows:

- Initially $map^+ = map^- = True$ since all of $\mathcal{P}(C)$ are unexplored.
- To mark a satisfiable set $N \subseteq C$ and all its subsets as explored we add to $map^+$ the clause $\bigvee_{i:c_i \notin N} x_i$.
- Symmetrically, to mark an unsatisfiable set $N \subseteq C$ and all its supersets as explored we add to $map^-$ the clause $\bigvee_{i:c_i \in N} \neg x_i$.

We use the SAT solver miniSAT [18] to hold and query the formulas $map^+$ and $map^-$. To get an arbitrary element of *Unexplored*, we can ask miniSAT for a model of $map^+ \wedge map^-$. However, in our algorithms, we need to be able to obtain two specific kinds of unexplored subsets.

First, given a set $N$, $N \subseteq C$, we need to be able to find a maximal unexplored subset of $N$. We exploit that miniSAT allows the user to fix values of some variables and also to set the default *polarity* of variables, i.e. the default value assignment to variables in decision points during the solving. To get a maximal unexplored subset of $N$, we fix the values of the variables $\{x_i | c_i \notin N\}$ to *False*, set the default polarity to *True*, and ask miniSAT for a model of $map^+ \wedge map^-$.

Second, given an *unexplored* $N$, $N \subseteq C$, we need to find a minimal unexplored subset $B$ of $N$ (this is used by TOME while constructing a chain of unexplored subsets). To do this, we fix the values of the variables $\{x_i | c_i \notin N\}$ to *False*, set the default polarity to *False*, and ask miniSAT for a model of $map^+$. Note that we do not include $map^-$ in the query. Intuitively, $map^-$ requires an absence of constraints and since $N$ satisfies $map^-$, every subset of $N$ also satisfies $map^-$.

As for the implementation, we integrate miniSAT via it's C API and we maintain two instances of the solver. One instance holds the formula $map^+ \wedge map^-$ whereas the other instance holds just $map^+$. Both the instances are used incrementally, i.e. the formulas are incrementally build during the whole MUS enumeration and simplified (internally by miniSAT) when possible. Let us note that Liffiton et al. also incrementally use miniSAT in their tool[1]. However, they maintain just the whole conjunction $map^+ \wedge map^-$ since a separate maintenance of $map^-$ or $map^+$ would not bring any speed-up in case of their MUS enumeration algorithm.

[1] https://sun.iwu.edu/%7eliffito/marco/

Finally, Explorer provides one more functionality. Given an unexplored subset $N$, Explorer can collect minable critical constraints of $N$. Recall that a constraint $c \in N$ is minable critical for $N$ iff $N \setminus \{c\}$ is explored. All the minable critical constraints can be determined based on the formula $map^+ \wedge map^-$. In particular, if we simplify the formula by fixing the variables $\{x_i | c_i \notin N\}$ to *False*, then values of some variables from $\{x_i | c_i \in N\}$ will be *implied* to be *True*. These implicants correspond to the minable critical constraints. This observation has been already exploited by Liffiton et al. [22] and they use miniSAT to obtain the implicants in their tool. However, the miniSAT's procedure for computing the implicants is not dedicated solely to this purpose; it is optimized w.r.t. the overall satisfiability solving process. Therefore, a use of miniSAT for this task brings an unnecessary overhead. In our tool, we directly compute the implicants from the formula $map^+$ instead of using a SAT solver to do it.

#### 4.3 SatSolver

*SatSolver* (declared in *SatSolver.h*) is an abstract class stating all the domain specific functionality that needs to be implemented (in a derived class) to support a particular constraint domain in our tool. There are three methods that have to be implemented by every derived class:

- `toString(`$N$`)` takes as an input a set $N$, $N \subseteq C$, and returns a textual representation of the constraints contained in $N$ (e.g. in the SMT-LIB 2 format if $N$ is a set of SMT constraints). We use this method to output the identified MUSes.
- `solve(`$N$`, ` $core = False$`, ` $extension = False$`)` takes as an input a subset $N$ of $C$ and returns *True* iff $N$ is satisfiable and *False* otherwise. Moreover, `solve` takes two optional Boolean parameters, *core* and *extension*, with default values set to *False*. If *core* is set to *True* and $N$ is unsatisfiable, `solve` also finds an *unsat core* of $N$, i.e. an unsatisfiable $M$ such that $M \subseteq N$. Similarly, if *extension* is set to *True* and $N$ is satisfiable, `solve` finds an *extension* of $N$, i.e. a satisfiable set $M$ such that $N \subseteq M \subseteq C$. We use the unsat cores in our tool to reduce seeds before shrinking. The extensions are used to further prune the set *Unexplored* when an unexplored subset is found to be satisfiable.
- `constructor(`*filepath*`)`. Every derived class of SatSolver has to implement its constructor. The constructor accepts a path *filepath* to a file that specifies the input set $C$ of constraints in some domain specific format (e.g. SMT-LIB 2 for SMT formulae). We invoke the constructor during the initialisation phase of our tool and its goal is to parse the input set of constraints and internally store the constraints for future manipulations. SatSolver is the only one of the six logical components of our tool that directly works with particular constraints of $C$. All the other components work just with a bitvector representation of subsets of $C$. For example, if $C = \{c_1, c_2, c_3, c_4\}$ is a set of four constraints and $K = \{c_1, c_2\}$, the bitvector representation of $K$ is 1100. Therefore, whenever another component communicates with SatSolver, e.g. invokes the procedure `solve(`$N$`)`, it passes the bit-vector representation of $N$ to SatSolver and SatSolver converts it to particular constraints.

Besides the above three methods that have to be implemented by every derived class, SatSolver defines and implements a method that can be overridden by a derived class:

- `shrink(`$N$`,` *crits*`)` performs the shrinking, i.e. it takes an unsatisfiable set $N$ together with a set *crits* of constraints that are critical for $N$ and returns a MUS of $N$. The default domain agnostic implementation of this method is carried out by Algorithm 1 (Section 2.2).

Currently, our tool supports 3 constraint domains via the following 4 derived classes of SatSolver:

- **MSHandle** (implemented in *MSHandle.cpp*) provides a functionality for the Boolean CNF domain, i.e. the set of constraints is a set of Boolean clauses. The input and output format is the DIMACS CNF format. For shrinking, we integrate two single MUS extraction tools: muser2 [5] by Belov and Silva, and a tool [1] by Bacchus and Katsirelos. Finally, we use miniSAT [18] to implement the method `solve`. Besides checking $N$ for satisfiability, we also use miniSAT to obtain an unsat core or an extension of $N$. In particular, an unsat core is directly provided by miniSAT. To get an extension of $N$, we obtain a model $\pi$ of $N$ from miniSAT and collect the set $\{c|c \in C \wedge \pi \models c\}$ of all constraints in $C$ that are satisfied by $\pi$.
- **Z3Handle** (implemented in *Z3Handle.cpp*) processes SMT constraints that are represented in the SMT-LIB2 format. We use z3 [16] to parse the input and to implement `solve`. Moreover, in the same way as in the case of MSHandle, we obtain unsat cores from z3 and we also obtain models of satisfiables formulas to compute their extensions. The shrinking is implemented using our custom procedure.
- **SpotHandle** (implemented in *SpotHandle.cpp*) supports the LTL domain. We use SPOT [17] to implement `solve` and the default domain agnostic implementation of `shrink`. In this case, we do not provide support for computing *non-trivial* unsat cores and *non-trivial* extension. Therefore, if an extension or unsat core is required while calling `solve(`$N$`)`, we simply use $N$ itself ($N$ is a trivial unsat core/extension of $N$).
- **NuxmvHandle** (implemented in *NuxmvHandle.cpp*) is another alternative for the LTL domain. Instead of SPOT, it uses nuXmv [11] as a satisfiability solver, which is, based on our experience, much more efficient than SPOT. However, nuXmv's license[2] is more restrictive than the SPOT's license and thus not every user of our tool might use it. In this case, we also do not support an extraction of non-trivial unsat cores and extensions.

If anyone wants to add support for another constraint domain to our tool, it is enough to implement a derived class of SatSolver. For example, the implementation of SpotHandle takes only 45 lines of code, including several empty lines

[2] https://es-static.fbk.eu/tools/nuxmv/index.php?n=Main.License caused by formatting and lines containing only closing brackets ("}"). Therefore, we claim our tool to be indeed domain agnostic and ready-to-use solution for any constraint domain.

#### 4.4 Installation and Execution of the Tool

For detailed installation and usage instructions, please follow the README.md file at: https://github.com/jar-ben/mustool.

Briefly, our tool can be built either in lightweight settings with support only for SAT domain, or with support also for the SMT and/or LTL domains. Whereas in the SAT domain, we use miniSAT that can be built very quickly, the z3 and SPOT solvers that we use in the SMT and LTL domains can take several hours to install. Once you have installed all the solvers you want to use, our tool can be simply built with an invocation of the command "make".

To run our tool in its default settings, execute:

`./must input_file`, where `input_file` specifies the input file of constraints, and it has to have either .cnf, smt2, or .ltl extension. Based on the extension, Master selects and uses an appropriate derived class of SatSolver. To specify a MUS enumeration algorithm to be used, invoke the tool by:

`./must -a alg input_file`, where `alg` can be either `marco`, `tome`, or `remus` (the default one). To see all the available settings, run

`./must -h`.

### 5 Experimental Evaluation

#### 5.1 Evaluated Tools

The only other existing MUS enumeration tool that can be seen as domain agnostic is the implementation[3] of the domain agnostic algorithm MARCO (invented by Liffiton et al. [22] and implemented by Liffiton and Zhao). In the following, we refer to the tool as `MARCO`. Currently, `MARCO` supports the SAT and SMT domains and can also relatively easily be extended to support another constraint domains. Here, we provide results of an experimental comparison of our tool `MUST` with `MARCO` in both the SAT and SMT domains. Moreover, to demonstrate that our domain agnostic tool can be competitive even to fully domain specific solutions, we include a comparison with two state-of-the-art MUS enumeration tools from the SAT domain: `MCSMUS`[4] [2] and `FLINT`[5] [25].

Due to the space limitation, we show here only results achieved by the best (default) configurations of our tool. In particular, in both domains, we use the

[3] https://sun.iwu.edu/%7emliffito/marco/
[4] https://bitbucket.org/gkatsi/mcsmus/src
[5] The tool was kindly provided to us by its author, Nina Narodytska.

algorithm ReMUS. As for the shrinking, in SMT domain, we use our custom shrinking solution, and in the SAT domain we employ a single MUS extraction algorithm by Bacchus and Katsirelos [1]. Complete results of the evaluation are available at: https://www.fi.muni.cz/%7exbendik/research/must.

All experiments were run using a time limit of 3600 seconds and computed on an Intel(R) Core(TM) i5-4690 CPU, 3.50GHz, 16 GB memory machine running Arch Linux 4.19.69-1-lts. The comparison criterion used in our evaluation is the number of identified MUSes within the given time limit.

#### 5.2 Benchmarks

In the SAT domain, we used a collection of 291 Boolean CNF benchmarks that were taken from the MUS track of the SAT 2011 Competition[6]. This collection has been used in many recent MUS related papers (e.g. [22,7,9,25,2]), including the ones that present `MARCO`, `FLINT`, and `MCSMUS`. The benchmarks range in their size from 70 to 16 million constraints and use from 26 to 4.4 million variables. In case of 28 benchmarks, all the evaluated algorithms identified all the MUSes within the given time limit. Since the comparison criterion of our evaluation is the number of identified MUSes, the 28 benchmarks are irrelevant for the evaluation (all three tools found the same number of MUSes for these benchmarks). Therefore, only the remaining 263 benchmarks are the subject of our evaluation.

In the SMT domain, we used a collection of 433 benchmarks that were taken from the QF_UF, QF_IDL, QF_RDL, QF_LIA and QF_LRA divisions of the library SMT-LIB[7]. Also this collection has been already used in several works, e.g. in the work by Cimatti et al. [13] or in our recent papers [9,8]. The benchmarks range in their size from 70 to 16 million constraints and use from 26 to 4.4 million variables. In case of 249 benchmarks, both the evaluated algorithms identified all the MUSes. Therefore, we focus here on the remaining 184 benchmarks.

#### 5.3 Results

In Figs. 2a, 2b, and 2c, we provide scatter plots that compare pair-wise `MUST` with the other tools in the SAT domain, and in Fig. 2d a scatter plot comparing `MUST` with `MARCO` in the SMT domain. Each point in a scatter plot corresponds to a single benchmark and shows the number of MUSes identified by the two algorithms. The x-coordinate of a point is given by the algorithm that labels the x-axis and the y-coordinate is given by the algorithm that labels the y-axis. Moreover, note that each scatter plot contains three additional numbers that are above/on right/in the right corner of the plot. These numbers show the number of points that are above/below/on the diagonal, respectively.

In the SMT domain, `MUST` conclusively dominates `MARCO`: it found more, less, and the same number of MUSes as `MARCO` in case of 100, 32, and 52 benchmarks, respectively. In the SAT domain, `MUST` outperforms on majority of benchmarks

[6] http://www.cril.univ-artois.fr/SAT11/
[7] http://www.smt-lib.org/

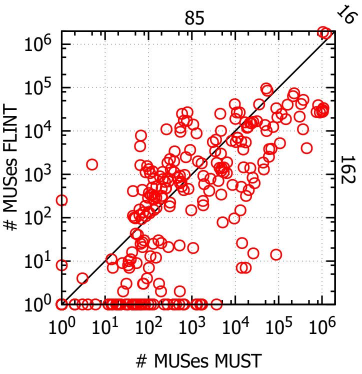

(a) MUST vs. FLINT, SAT domain

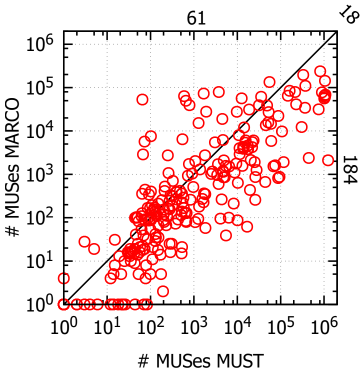

(b) MUST vs. MARCO, SAT domain

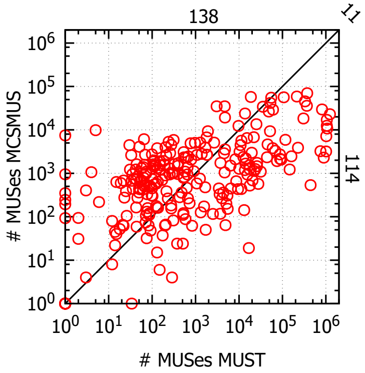

(c) MUST vs. MCSMUS, SAT domain

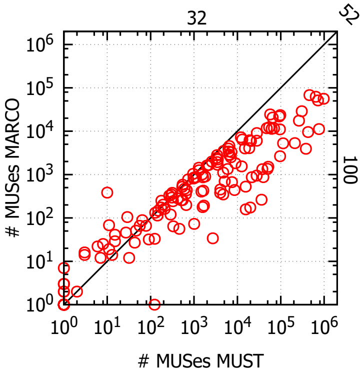

(d) MUST vs. MARCO, SMT domain

Fig. 2: Scatter plots comparing the number of produced MUSes.

both MARCO and FLINT. Finally, MCSMUS outperforms MUST in case of 52 percent of benchmarks and is worse than MUST in case of 43 percent of benchmarks. Still, this is a very good result since MUST is a domain agnostic tool whereas MCSMUS is tailored to the SAT domain.

Besides the pair-wise comparison of the algorithms, we also provide an overall ranking of the algorithms on individual benchmarks in the SAT domain. In particular, assume that for a benchmark B both MUST and MCSMUS found 100 MUSes, FLINT found 80 MUSes, and MARCO found 50 MUSes. In such a case, MUST and MCSMUS share the 1st (best) rank for B , FLINT is 3rd, and MARCO is on the 4th position. In Fig. 3 we show the average ranking (from all benchmarks) of all algorithms for each subsequent 60 seconds of the computation. We can see that MARCO ranked the worse during the whole computation. FLINT ranked quite well during the first 600 seconds, but then its performance degraded. Finally, MUST and MCSMUS maintained the best and the second best ranking, respectively. This might be quite surprising since MCSMUS is slightly better than MUST in Fig. 2c.

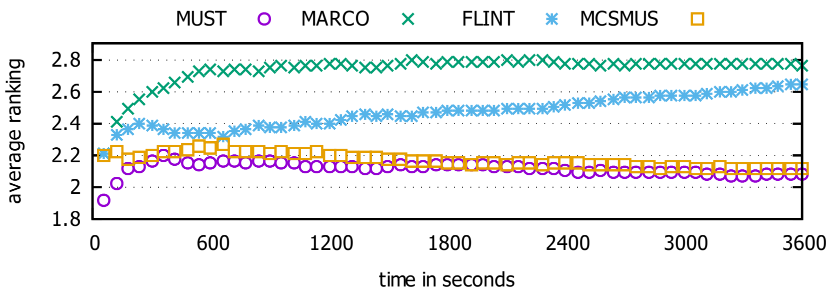

Fig. 3: Average ranking in time.

The thing is that MUST mostly ranks either as 1st or 2nd on a benchmark and rarely ranks as 4th, whereas MCSMUS more often ranks as 3rd or 4th.

Finally, let us recall that our tool contains also implementation of the algorithm MARCO and thus one might be interesting in comparing the performance of MARCO in our tool and MARCO in the tool MARCO. In the SAT domain, we found our implementation to be more efficient, equal, and less efficient than MARCO in case of 68, 6, and 26 percent of benchmarks, respectively. In the SMT domain, our implementation is better, equal, and worse in 37, 29, 34 percent of benchmarks, respectively[8]. Therefore, shall anyone want to use the algorithm MARCO, we recommend to use our implementation.

### 6 Case Study

During the last 4 years, we participated on the European Union's Horizon 2020 project called AMASS [26]. The project brought together researchers from academia and engineers from large industrial companies such as Honeywell, Alstom, or Infineon. The project focused on improving the process of development and certification of Cyber-Physical Systems in markets such as automotive, railway, aerospace, space, and energy. Among others, this included the development of techniques for assessing quality of system specification/requirements and this is where our tool found an application.

Establishing the requirements is an important stage in all development. In general, the requirements can be expressed either informally, e.g. using a natural language, or formally by employing a kind of mathematical logic such as the Linear Temporal Logic (LTL). The formalization removes ambiguity and allows to employ various model-based techniques, such as model checking. Moreover, we get the opportunity to verify the requirements earlier, even before any system model is built. In particular, we can verify that the requirements are consistent (satisfiable), i.e. that there can be even built a system that satisfies all the requirements. If the requirements are inconsistent, they need to be refined.

[8] See the appendix https://www.fi.muni.cz/%7exbendik/research/must

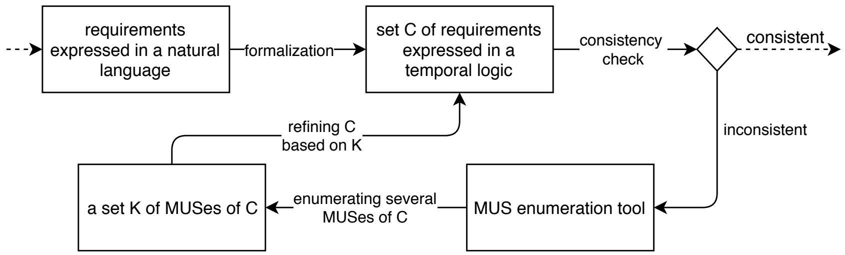

Fig. 4: Application of MUS enumeration in requirements analysis.

Within the AMASS project, we proposed a scheme [6] that exploits MUSes to help the user to establish a consistent set of requirements. A basic workflow of the scheme is depicted in Fig. 4. The process starts by introducing a set of requirements in some natural-language like format, yet using a restricted grammar that avoids ambiguities. In the next step, the requirements are formalized using LTL and gathered in a set $C$. Subsequently, $C$ is checked for consistency. If $C$ is consistent, then the software development process can continue with a next stage. Otherwise, a MUS enumeration tool is used to identify a set $K$ of MUSes of $C$, and the user uses $K$ to refine $C$. The MUS identification and refinement steps are repeated until the set of requirements becomes consistent.

We implemented the scheme in AMASS as a part of a so-called V&V manager [27]: a tool for validation and verification of the system model and system requirements. Our industrial partners employed the scheme on a set of industrial benchmarks, and evaluated two contemporary MUS enumeration tools from the LTL domain: our `MUST`, and `Looney` by Bauch et al. [4]. They found `MUST` to be faster by several orders of magnitude. Unfortunately, the industrial benchmarks are confidential and cannot be published in this paper. Yet, authors of `Looney` indeed acknowledge in their paper that `Looney` can handle only small input constraint sets containing just low tens of constraints. On the other hand, `MUST` was shown [8] to be able to efficiently work with hundreds of constraints.

### 7 Conclusion

We presented a tool, called MUST, for online enumeration of Minimal Unsatisfiable Subsets (MUSes). MUST implements three contemporary *domain agnostic* MUS enumeration algorithms, i.e. algorithms that can be applied in any constraint domain. Currently, the tool supports enumeration in the SAT, SMT and LTL domains, and can be easily extended to support another domains. Therefore, we classify the tool itself as *domain agnostic*; it serves as (an almost) ready-to-use solution for any domain where MUSes already find or eventually will find an application. We experimentally compared MUST to a domain agnostic tool by Liffiton et al. [22] in the SAT and SMT domains, and we showed that MUST conclusively dominates in both domains. Moreover, we showed that MUST is even competitive to contemporary tools that are tailored for the SAT domain.
